# Cost–Benefit Optimization of Structural Health Monitoring Sensor Networks

**DOI:** 10.3390/s18072174

**Published:** 2018-07-06

**Authors:** Giovanni Capellari, Eleni Chatzi, Stefano Mariani

**Affiliations:** 1 Politecnico di Milano, Dipartimento di Ingegneria Civile e Ambientale, Piazza Leonardo da Vinci 32, 20133 Milano, Italy; stefano.mariani@polimi.it; 2 ETH Zürich, Institut für Baustatik und Konstruktion Stefano-Franscini-Platz 5, 8093 Zürich, Switzerland; chatzi@ibk.baug.ethz.ch

**Keywords:** structural health monitoring, Bayesian inference, cost–benefit analysis, stochastic optimization, information theory, Bayesian experimental design, surrogate modeling, model order reduction

## Abstract

Structural health monitoring (SHM) allows the acquisition of information on the structural integrity of any mechanical system by processing data, measured through a set of sensors, in order to estimate relevant mechanical parameters and indicators of performance. Herein we present a method to perform the cost–benefit optimization of a sensor network by defining the density, type, and positioning of the sensors to be deployed. The effectiveness (benefit) of an SHM system may be quantified by means of information theory, namely through the expected Shannon information gain provided by the measured data, which allows the inherent uncertainties of the experimental process (i.e., those associated with the prediction error and the parameters to be estimated) to be accounted for. In order to evaluate the computationally expensive Monte Carlo estimator of the objective function, a framework comprising surrogate models (polynomial chaos expansion), model order reduction methods (principal component analysis), and stochastic optimization methods is introduced. Two optimization strategies are proposed: the maximization of the information provided by the measured data, given the technological, identifiability, and budgetary constraints; and the maximization of the information–cost ratio. The application of the framework to a large-scale structural problem, the Pirelli tower in Milan, is presented, and the two comprehensive optimization methods are compared.

## 1. Introduction

Structural health monitoring (SHM) allows the detection and estimation of variations in the behavior and thereby condition of engineered systems [1], and therefore helps in making decisions about the actions needed to maintain or recover the overall structural safety [2]. Amongst the available methods for SHM in the literature (see [1]), the Bayesian framework allows both unknown system properties and their associated uncertainties to be estimated, as introduced in [3]. The effectiveness of any SHM system to estimate and detect damage, here assumed as a variation of mechanical properties, depends on both the estimation method exploited to crunch the data and the SHM system itself. From a theoretical point of view, we can interpret SHM as an experimental procedure, where the measurements obtained through the SHM system are exploited to reach the goal of the experiment (i.e., the estimation of the parameters and their associated uncertainties). In this view, the experimental setup in SHM includes all the settings which can affect the measurements—namely, the position of the sensors on the structure, the physical quantities to be measured, the number of sensors, and the type of sensors.

In the present work, a SHM system cost–benefit optimization method is presented, which allows the most suitable experimental settings to be chosen in order to maximize the estimation potential and simultaneously minimize the cost of the SHM system. Two alternative approaches are proposed and discussed: (i) the system is optimized by maximizing its effectiveness and simultaneously fulfilling the budgetary constraint; (ii) the system is optimized by maximizing the ratio between its effectiveness and its cost.

Two main advantages can be derived from optimizing the sensor network. Let us first assume that by optimizing the sensor network in terms of position and types of sensors, their number can be decreased. This results in a cost reduction of the overall SHM system and a simplification of the data acquisition system and the system assembly phase. Moreover, an additional side benefit lies in the reduction of the amount of data to be processed. In other words, the resulting optimized SHM system is more “informative”, and thus fewer sensors are required to guarantee the same accuracy of the estimated quantities. Consequently, both the cost and the complexity of the data storage system and the required computational resources can be significantly reduced. Moreover, since the amount of data to be processed is lower, the applicability of real-time estimation methods is enhanced, and the required data storage is reduced for non-real-time applications. On the contrary, if the number and type of sensors are assumed to be constant, the optimization of the sensor network guarantees an increase of information provided by the monitoring system, and thus a consequent reduction of the estimate uncertainties.

Several methods have been presented in the literature to optimally design SHM systems: in all of them, the type and number of sensors are assumed to be constant. The vast majority of these prescribes the type and number of sensors as constant parameters in the optimization problem, allowing for the optimization of the spatial configuration alone. Among them, a stochastic approach for the optimal sensor placement, based on the minimization of a Bayesian loss function, was introduced in [4]: the sensor locations were chosen such that the expectation of the squared error loss function between the estimated and the target values of the quantities to be estimated was minimized. In [5], the evaluation of entropy and mutual information was proposed in order to quantify the amount of information, which can be inferred experimentally (and therefore from an SHM system).

In [6,7], Papadimitriou et al. proposed the minimization of the information entropy as a rationale to optimize the spatial configuration of the sensor network. In order to numerically evaluate the associated objective function, the integral terms arising from an analytical manipulation of the information entropy were approximated through the Laplace method of asymptotic expansions, which allows an associated algebraic formulation to be obtained. Unlike in the present study, the objective function was locally approximated through a smooth replica centered on nominal parameter values, which have to be chosen a priori.

The optimization algorithm to obtain the optimal solution (i.e., maximization of the objective function) has mostly been treated as a discrete optimization problem in the existing literature, with genetic algorithms exploited in [8,9]. According to an alternative greedy approach proposed in [7,10], the optimal configuration can be obtained by splitting the optimization problem into a number of sub-problems, where only one sensor is added at each step, so that the increase in the objective function value is maximized.

In contrast to the existing methods, in the present work a method is introduced to comprehensively optimize the sensor network not only in terms of sensor placement, but also in terms of the type and number of sensors. The effectiveness of a sensor network is quantified through an index based on information theory, originally developed within the computer science research community for the quantification of uncertainty relating to random variables. The SHM sensor network is therefore optimized in terms of number, position, and type of sensors by maximizing the relevant expected Shannon information gain, which is a measure of the utility of the measurements with respect to the quantities to be estimated. It should be underlined that the proposed method was developed within a Bayesian framework and is therefore valid for SHM procedures aiming at Bayesian inference, such as Bayesian model updating or parameter characterization. It can be applied to processes that run both offline and in real-time (as in the reasoning of Kalman filtering-based estimation [11,12,13,14]). As in most stochastic approaches, large computational resources would be needed to take the uncertainties into account, preventing the applicability of the method to large structural models. The coupling with surrogate models (polynomial chaos expansion, [15]), which aims at replacing the original computationally expensive numerical model by reproducing the relation between inputs and outputs, and model order reduction strategies (principal component analysis) allow the latter problem to be overcome. The use of stochastic optimization methods (covariance matrix adaptation-evolutionary strategy, [16]) allows high-dimensional problems to be solved, which was not possible with the previously exploited methods.

The method developed herein is applicable to both static and dynamic monitoring applications of diverse sensing capabilities, where a Bayesian framework is intended to be implemented. The method is demonstrated herein for static measurements only. However, the same framework can be implemented for dynamic monitoring as well if the objective function is expressed in terms of frequency and mode-shape matching.

This paper is organized as follows: first, the theoretical framework is presented in Section 2 by introducing the Bayesian experimental design in Section 2.1 and defining the optimization statement in Section 2.2. Then, the approach for numerically evaluating the objective function is discussed in Section 3 and Section 4. In Section 5, the whole optimization procedure is summarized. Then, the application of the method to a tall building, namely the Pirelli tower in Milan, is presented in Section 6. Finally, some concluding remarks are gathered in Section 7.

## 2. Theoretical Basis

### 2.1. Bayesian Experimental Design

Let the goal of the SHM system be the estimation of a set of parameters (e.g., mechanical properties, geometrical properties, or damage indices) defined within an appropriate numerical model of a structure, used to predict its response to given loads. The following random vectors are defined: the parameter vector $\theta =\left[\theta_{1}\;\theta_{2}\cdots \;\theta_{n_{\theta }}\right]\in R^{n_{\theta }}$ to be estimated; the data vector $y\in R^{n_{y}}$, with measurements assumed to be collected through a set of sensors. Here, $n_{\theta }$ is the number of parameters and $n_{y}$ is the number of measurements.

The prior probability density function (pdf) p(θ) represents the prior knowledge on θ, and it can be suitably chosen in order to take initial information into account, such as that provided by previous experiments or by the subjective belief of an expert. If no previous information is available on the values of parameters in θ, an uninformative distribution can be considered. The pdf may then be updated considering the data y, through Bayes’ theorem:

(1) $$p\left(\theta |y\right)=\frac{p(y|\theta )p(\theta )}{p(y)},$$

where the expression p(·|·) represents the conditional pdf of the first term with respect to the second one. Thus, p(θ|y) is the posterior pdf (i.e., the probability density function of θ), given y; p(y|θ) is the likelihood; p(y) is the evidence, that is, the distribution of the observed data, marginalized over  θ.

Bayes’ theorem is particularly suited within such a context in SHM problems: given a structure, a class of models can be a-priori defined to describe the behaviour of the system, and updated as soon as the structure response is measured. Bayesian model updating was first introduced for structural applications in [3,17], and it allows the posterior probability density function p(θ|y) to be obtained based on prior knowledge of θ and the data, that is, the maximum a posteriori estimate $\theta^{*}=\arg\max_{\theta }\left[p\left(\theta |y\right)\right]$, along with the related uncertainty level.

Within this framework, the effectiveness of the sensor network can be quantified by following the decision-theoretic approach introduced in [18,19]: in the case at hand, prior to performing the measurements, the choice of the experimental settings—in terms of spatial sensor configuration, number of sensors, and type of sensors—must be made. An additional term—the design variable $d\in R^{n_{d}}$, with $n_{d}$ designating the dimension of the design variable vector—must be accordingly introduced in the formulation in order to parametrize the network topology and the sensor features. Bayes’ theorem in Equation (1) is then modified as:

(2) $$p\left(\theta |y,d\right)=\frac{p(y|\theta ,d)p(\theta |d)}{p(y|d)},$$

where all the previously introduced pdfs are conditioned with respect to the design variable d, as both the measurements and the parameters to be estimated depend on the experimental settings. Here, the unknown variable d is supposed to define the spatial configuration of the sensors (e.g., in terms of spatial coordinates), for a constant number of measurements $n_{y}$ and type of sensors.

According to [18], the expected utility of one experiment can be quantified through:

(3) $$U\left(d\right)=\int_{Y}\int_{\Theta }u\left(d,y,\theta \right)p\left(\theta |y,d\right)p\left(y|d\right)d\theta dy,$$

where Y and Θ respectively represent the domains of the measurements y and of the parameters θ.

The function u(d,y,θ) is called utility function and defines a scalar measure of the usefulness of the experiment. That is, it quantifies the extent to which certain measurement values are preferable to attain the goal of SHM.

Within a stochastic environment and from a decision-theory perspective, the expected utility allows to choose which action should be performed in order to achieve a certain goal. Therefore, it can be defined as the weighted average of the utilities of each possible consequence of a certain action, wherein the weights describe the probabilities that an action would lead to a certain outcome. For SHM applications, the action is represented by the design of the monitoring system, and the goal is the estimation of the unknown structural parameters.

The choice of u(d,y,θ) depends on the goal of the experiment. A thorough review of utility functions is presented in [20]. In the present case, the aim of the experiment is the inference (estimation) of the parameters θ. Therefore, following [21], a suitable utility function is the Kullback-Leibler divergence (KLD) [22,23] (also called relative entropy) between the prior and the posterior pdfs. Supposing that the structural response to loading is linear and the posterior pdfs are Gaussian, the optimization problem results in the so-called Bayesian D-optimality [24], which corresponds to the maximization of the determinant of the Fisher information matrix [25] of the measurements.

The KLD from *P* to *Q*, which are two generic probability distributions of a random variable x, is defined as:

(4) $$D_{KL}\left(P||Q\right)=\int_{X}p\left(x\right)\ln\frac{p(x)}{q(x)}dx,$$

where X is the domain of x; p(x) and q(x) are the pdfs related to *P* and *Q*. The KLD thus measures the increase of information from *Q* to *P*. If the two distributions are identical (i.e., P=Q almost everywhere), then $D_{KL}\left(P||Q\right)=0$.

If the goal of the Bayesian inference problem is the design of the sensor network such that the most possible information is provided by the measurements y on the parameters θ to be estimated, the design variable d has to be optimized by maximizing the gain between the prior pdf p(θ|d) and the posterior pdf p(θ|y,d). By specializing Equation (4) for the problem considered here, the resulting utility function is given as:

(5) $$u\left(d,y,\theta \right)=D_{KL}\left[p(\theta |y,d)||p(\theta |d)\right]=\int_{\Theta }p\left(\theta |y,d\right)\ln\frac{p(\theta |y,d)}{p(\theta |d)}d\theta .$$

It should be noted that in the integral of Equation (5) the parameter vector θ serves as a dummy variable. Therefore, u(d,y,θ) is not a function of θ. Thus, the expected utility function in Equation (3) can be written as:

(6) $$\begin{array}{c} U\left(d\right)=\int_{Y}\int_{\Theta }p\left(\theta |y,d\right)\ln\frac{p(\theta |y,d)}{p(\theta |d)}p\left(y|d\right)d\theta dy, \end{array}$$

where U(d) is called the expected Shannon information gain [26] or the Lindley information measure [18].

### 2.2. Optimal Design of the SHM System

In order to provide a comprehensive strategy to optimize the sensor network, the number of measurements $n_{y}$ and the pdf $p_{\epsilon }$ of the so-called prediction error $\epsilon \in R^{n_{y}}$ are taken into account as unknown variables of the relevant optimization problem.

Let the prediction error ϵ be sampled from a zero mean Gaussian noise $p_{\epsilon }=\;N\left(0,\Sigma \right)$, where Σ is the covariance matrix (however, in principle the proposed method can be applied to any kind of $p_{\epsilon }$). The expected Shannon information gain then generally depends on the sensor configuration, number of measurements, and prediction error (i.e., $U=U(d,n_{y},\Sigma )$).

Within this framework, the prediction error accounts for the measurement errors related to the sensor characteristics and the model error associated with the intrinsic numerical approximations. Assuming independence between these two error sources, the covariance matrix can be written as:

(7) $$\Sigma =\Sigma_{m}+\Sigma_{n},$$

where $\Sigma_{m}$ and $\Sigma_{n}$ respectively account for the model and the measurement error. In [27], it has been shown that the optimal sensor configuration can also be affected by the spatial correlation among different measurements, which can be taken into account in $\Sigma_{m}$. In practice, the correlation between any couple of measurements decays exponentially with the distance between their locations. A spatial correlation length can be then introduced to constrain the optimal spatial configuration. $\Sigma_{n}$ can be instead related to the type of sensors to be employed in the SHM system—that is, to the instrumental noise which depends on their characteristics (e.g., signal-to-noise ratio).

For the sake of simplicity, it is assumed that: the sensor type is unique, and so the measurement noise can be accounted for through $\Sigma_{n}=\sigma^{2}I$; and the model error $\Sigma_{m}$ is treated as a constant that can affect the optimal configuration, but it is not handled as a further object of the optimization procedure. The resulting optimization statement thus reads:

(8) $$\left(d^{*},n_{y}^{*},\sigma^{*}\right)=\arg\max\left[U(d,n_{y},\sigma )\right].$$

Three types of constraints must be taken into account in the problem:

(a) identifiability constraint: $n_{y}>n_{iden}$, where $n_{iden}$ is the minimum number of measurements which are required in order to guarantee the identifiability of the parameters θ (see [17,28,29]);
(b) technological constraint: $\sigma >\sigma_{sens}$, where $\sigma_{sens}$ is the lowest standard deviation of the measurement noise, associated with the sensors available on the market and chosen to measure the structural output;
(c) cost constraint: $C(n_{y},\sigma )\leq B$, where $C(n_{y},\sigma )$ is the cost model of the SHM system and *B* is the maximum budget available for SHM.

The whole optimization problem can therefore be stated as:

(9) $$\begin{array}{c} \left(d^{*},n_{y}^{*},\sigma^{*}\right)=\arg\max\left[U(d,n_{y},\sigma )\right],\\ \mathrm{subject}\;\mathrm{to}\;\;\left\{\begin{array}{c} n_{y}>n_{iden},\\ \sigma >\sigma_{sens},\\ C(n_{y},\sigma )\leq B. \end{array}\right. \end{array}$$

Regarding the cost model $C(n_{y},\sigma )$, the simplest possible consists of a combination of a sensor network cost $C_{0}$, which for example includes the data acquisition hardware, database, assemblage, etc., plus a variable cost (i.e., the cost of all the sensors to be deployed over the structure). Accordingly:

(10) $$C\left(n_{y},\sigma \right)=C_{0}+c\left(\sigma \right)\;n_{y},$$

where c(σ) is the unitary cost per sensor.

In order to solve the optimization problem, a possible approach would be to embed the unknown variables $n_{y}$ and σ into the design variable vector d. An alternative approach, which is particularly suitable for real applications if only a limited set of sensor types is available, is to explore the function $\bar{U}=U\left(d^{*},n_{y},\sigma \right)$ [30], which represents the maximum of the expected Shannon information gain over a search grid of points $\left\{n_{y},\sigma \right\}$. $d^{*}$ is then the optimal configuration obtained by solving the relevant optimization statement, with fixed values of $n_{y}$ and σ. Since $d^{*}$ depends on the choice of $\left(n_{y},\sigma \right)$, it is possible to conclude that the function $\bar{U}=\bar{U}\left(n_{y},\sigma \right)$ depends exclusively on $\left(n_{y},\sigma \right)$.

In place of the preceding formulation, based on budget constraint, a procedure based on a cost–benefit analysis can be followed (see [31,32]). In the problem at hand, the benefit is represented by the expected Shannon information gain. Although $U(d,n_{y},\sigma )$ cannot be directly converted into an expected monetary gain (benefit), it is possible to define a utility–cost index (UCI) through [33]:

(11) $$\mathrm{UCI}\left(d,n_{y},\sigma \right)=\frac{U(d,n_{y},\sigma )}{C(n_{y},\sigma )},$$

whose measurement unit is [nat/€], [nat] standing for the natural unit of information. The associated optimization problem would then be:

(12) $$\begin{array}{c} \left(d^{*},n_{y}^{*},\sigma^{*}\right)=\arg\max\left[\frac{U(d,n_{y},\sigma )}{C(n_{y},\sigma )}\right],\\ \mathrm{subject}\;\mathrm{to}\;\;\left\{\begin{array}{c} n_{y}>n_{iden},\\ \sigma >\sigma_{sens},\\ C(n_{y},\sigma )\leq B. \end{array}\right. \end{array}$$

This optimization formulation allows the most efficient SHM design to be obtained (i.e., to maximize the information per unitary cost).

The same considerations reported previously hold for the optimization problem in Equation (12). The optimal solutions can be obtained by maximizing the associated objective function $\bar{UCI}=\mathrm{UCI}\left(d^{*},n_{y},\sigma \right)$, where $d^{*}$ is the optimal spatial configuration for each $\left\{n_{y},\sigma \right\}$ set in the search grid.

A comparison between the results of the two strategies defined in Equations (9) and (12) is discussed in Section 6. It is important to underline now that, because the measurements y depend on the loading conditions, the optimal sensor placement depends on them as well. Therefore, in order to obtain a sensor network design which is robust with respect to the input loading, several optimizations under different loads should be performed, and the final sensor network configuration should be chosen as the one providing the maximum value of the objective function or, alternatively, as a kind of envelope of all the available solutions.

## 3. Numerical Approach

As explained in Section 2.1, the optimal design of the SHM system is obtained by maximizing the expected Shannon information gain $U(d,n_{y},\sigma )$ (see Equation (9)), or a function related to it (see Equation (12)). At given values of $n_{y}$ and σ, the optimal experimental design $d^{*}$ defines the spatial configuration of the network for which the utility is maximized:

(13) $$d^{*}=\arg\max_{d\in D}\left[\int_{Y}\int_{\Theta }p\left(\theta |y,d\right)\ln\frac{p(\theta |y,d)}{p(\theta |d)}p\left(y|d\right)d\theta dy\right]=\arg\max_{d\in D}\left[U(d)\right]$$

D being the design space, which is the domain of all the possible experimental settings (e.g., the locations where the sensors can be placed).

Because the experimental design has to be put in place before performing the measurements, the optimal solution $d^{*}$ cannot be found by simply maximizing U(d,y,θ) with respect to y and θ, which are random variables. The optimal point is instead looked for in the design space D, by exploring the probability distributions p(θ|y,d) and p(y|d) in the domains Y and Θ.

In order to solve the optimization problem, a strategy to compute U(d) is needed. Since the double integration in Equation (6) generally cannot be performed analytically, a numerical procedure has to be adopted. Following [34,35] and assuming that p(θ|d)=p(θ) (i.e., that the prior distribution is independent of the design variable), Equation (6) can be approximated through the associated Monte Carlo (MC) estimator:

(14) $$\hat{U}\left(d\right)=\frac{1}{N_{out}}\sum_{i=1}^{N_{out}}\left\{\ln\left[p(y^{i}|\theta^{i},d)\right]-\ln\left[p(y^{i}|d)\right]\right\},$$

where $N_{out}$ is the number of samples $\theta^{i}$ and $y^{i}$ to be respectively drawn from p(θ) and $p(y|\theta =\theta^{i},d)$.

The term $p(y^{i}|d)$ can be computed through an analogous MC estimator as:

(15) $$p\left(y^{i}|d\right)≃\frac{1}{N_{in}}\sum_{j=1}^{N_{in}}p\left(y^{i}|\theta^{j},d\right),$$

where $N_{in}$ is the number of samples $\theta^{j}$ to be drawn from p(θ).

The computational cost of such an MC approach can be reduced by using the same batch of samples $\theta^{i}=\theta^{j}$ in Equations (14) and (15). The resulting number of likelihood function evaluations then decreases from $N_{in}\times N_{out}$ to $N=N_{in}=N_{out}$ (see [34]). The MC estimator of $\hat{U}\left(d\right)$ is then obtained as:

(16) $$\hat{U}\left(d\right)=\frac{1}{N}\sum_{i=1}^{N}\left\{\ln\left[p(y^{i}|\theta^{i},d)\right]-\ln\left[\frac{1}{N}\sum_{j=1}^{N}p\left(y^{i}|\theta^{j},d\right)\right]\right\}.$$

### Model Response

In Equation (16), a major issue is represented by the evaluation of the likelihood function $p(y^{i}|\theta^{j},d)$. Let the structural system, whose SHM network has to be designed, be subjected to a set of forces and constraints. Since its response to the loads depends on the unknown parameters θ, the measurements can be linked to the design variables in accordance with:

(17) y=L(d)v(θ)+ϵ=M(d,θ)+ϵ,

where $M\left(d,\theta \right):R^{n_{d}}\times R^{n_{\theta }}\to R^{n_{y}}$ is the forward model operator which relates the model inputs (i.e., the design variables d and θ) with the measurements y under the considered loading, $L\in R^{n_{y}}\times R^{n_{dof}}$ is a Boolean operator which aims at selecting from v the actually measured response components, $n_{dof}$ is the number of degrees of freedom (DOFs) of the numerical model, and $v\in R^{n_{dof}}$ is the structural response (e.g., displacements, rotations, etc.) of the numerical model for all the $n_{dof}$ degrees of freedom.

Following [28], the likelihood function can then be expressed as:

(18) $$p\left(y^{i}|\theta^{j},d\right)=p_{\epsilon }\left(y^{i}-M\left(d,\theta^{j}\right)\right).$$

It can be underlined that the same approach can be applied to dynamic cases as well. To this end, only Equation (17) has to be modified as follows:

(19) y=L(d)Φ(θ)+ϵ,

where $\Phi \in R^{n_{dof}}\times R^{N_{m}}$ is a matrix containing $N_{m}$ modal shapes of the structure and y are the relevant measurements. Equation (18) is accordingly changed to [28]:

(20) $$p\left(y^{i}|\theta^{j},d\right)=\prod_{m=1}^{N_{m}}p_{\epsilon }\left(y^{i}-L\left(d\right)\Phi_{m}\left(\theta^{j}\right)\right).$$

Apart from the definition of the likelihood function in Equation (20), the rest of the framework also remains valid for the dynamic case. Since this application goes beyond the scope of the present paper, future work will be devoted to the implementation of such an approach to dynamic testing.

Returning to the static case and considering Equations (16) and (18) and knowing that $y^{i}=M\left(d,\theta^{i}\right)+\epsilon^{i}$ (see Equation (17)), the MC estimator of the expected Shannon information gain is obtained as:

(21) $$\begin{array}{c} \hat{U}\left(d\right)=\frac{1}{N}\sum_{i=1}^{N}\left\{\ln\left[p_{\epsilon }\left(\epsilon^{i}\right)\right]\right\}-\frac{1}{N}\sum_{i=1}^{N}\left\{\ln\left[\frac{1}{N}\sum_{j=1}^{N}p_{\epsilon }\left(M\left(d,\theta^{i}\right)+\epsilon^{i}-M\left(d,\theta^{j}\right)\right)\right]\right\}. \end{array}$$

The MC estimator is thus offered as the sum of two terms: the first one depends on the prediction error only; the second one depends on the design variables as well as on the parameters.

If the prediction error ϵ can be assumed independent of the design variable d (i.e., if p(θ|d)=p(θ)), the first term in Equation (21) turns out to be independent of d. Therefore, it can be dropped from the computation, as we are interested only in the design vector $d^{*}$ providing the optimum. In this way, the computing time required for the evaluation of the objective function would be significantly reduced. This case occurs, for example, if a unique type of sensor is planned to be installed on the structure. If the standard deviation of measurements depends on the design variable (i.e., if σ=σ(d)), the first term must be kept in the objective function because it affects the optimal solution.

## 4. Surrogate Modeling

The computational cost of the MC estimator in Equation (21), as is true of any other MC analysis, may be attributed to the repeated evaluation of the model response $M(d,\theta^{i})$, for each of the *N* samples $\theta^{i}=\theta^{j}$ drawn from p(θ). From a practical point of view, the computation of $\hat{U}\left(d\right)$ can become infeasible due to the high number of degrees of freedom (DOFs) in the numerical models (e.g., of real-life structures).

To reduce the overall computational costs of the evaluation of the model response in the optimization procedure, the exploitation of surrogate models has been proposed in [35] and applied to SHM sensor network optimization in [36]. A surrogate model (or metamodel) is aimed at providing the relationship between input and output through a more computationally efficient formulation. These approaches can therefore be classified as data-driven ones: the underlying physics of the problem is lost. Accordingly, if the physical behavior of the problem changes, a new surrogate model should be built by using the new relevant input–output data. Alternative methods to reduce the computational cost of model evaluation are model-based (e.g., [37,38]).

One of the most widely exploited types of surrogate models is based on polynomial chaos expansion (PCE). PCE was first introduced in [15,39] for standard Gaussian random variables, and was then generalized to other probability distributions in [40,41,42].

Following the investigation in [43], it is assumed that the input random vector is constituted by the unknown parameters only, featuring a joint pdf p(θ), whereas the design variable d is not considered in the surrogate model.

Assuming a finite variance model, the PCE of the response v in Equation (17) reads:

(22) $$v_{k}=M_{k}\left(\theta \right)=\sum_{\alpha \in N^{M}}\phi_{\alpha }\Psi_{\alpha }\left(\theta \right)\;\;\;\;\;\;\;\;\;\;k=1,\ldots ,n_{dof},$$

where $\Psi_{\alpha }$ are multivariate polynomials which are orthonormal with respect to p(θ); $\alpha =\left\{\alpha_{1},\ldots ,\alpha_{M}\right\}\in N^{M}$ is a multi-index associated with the components of Ψ, and $\phi_{\alpha }\in R$ are the related coefficients. For real-life applications, the sum in Equation (22) is truncated by retaining only those polynomials whose total degree |α| is less than a certain value *p*:

(23) $$v_{k}≃M_{k}^{PCE}\left(\chi \right)=\sum_{|\alpha |\leq p}\phi_{\alpha }\Psi_{\alpha }\left(\theta \right)\;\;\;\;\;\;\;\;\;\;k=1,\ldots ,n_{dof},$$

where $\left|\alpha \right|=\sum_{i=1}^{M}\alpha_{i}$ and $M_{k}^{PCE}$ is the surrogate model. The response v can be approximated in a component-wise fashion by building a set of $n_{y}$ PCE surrogate models according to:

(24) $$v≅M^{PCE}\left(\theta \right)=\left\{\begin{array}{c} M_{1}^{PCE}\left(\theta \right)\\ \vdots \\ M_{n_{dof}}^{PCE}\left(\theta \right) \end{array}\right\}.$$

In order to compute the unknown polynomial coefficients $\phi_{\alpha }$ for each surrogate, both intrusive and non-intrusive methods can be adopted [44]. Intrusive approaches rely on the projection of the original computational model onto the subspace spanned by the PCE, through a Galerkin projection [45]. In such methods, the variables in the governing equations are replaced by their polynomial chaos expansions. For instance, for linear structural problems, the stiffness matrix and the response vector are approximated through a truncated expansion, leading to a linear system of equations to be solved [46]. While these methods demonstrate an increase of the computational cost which is linear with the number of basis polynomials, they are not suitable for the current purposes, since they require the custom modification of the computational solver.

Non-intrusive methods instead allow the bases to be computed by simply processing a batch of sampled input variables θ and the corresponding model evaluations v, which form the so-called experimental design. No manipulations of the solver are needed, rendering this approach particularly suitable for general-purpose problems. Two methods can be used for non-intrusively computing the coefficients: the projection approach [47,48], where the computation of each coefficient is formulated as a multi-dimensional integral; and least-squares minimization [49].

The latter method is employed here, since an arbitrary number of samples can be used in order to estimate the coefficients of the expansion in Equation (23). The corresponding formulation of the least-square minimization problem is:

(25) $$\hat{\phi }=\arg\;\min_{\phi }\;E\left\{{\left[\phi^{T}\Psi \left(\chi \right)-M\left(\chi \right)\right]}^{2}\right\},$$

where $\hat{\phi }$ is the whole set of coefficients to be estimated. In order to reduce the computational cost of the least-squares approach, a method based on least angle regression introduced in [50,51] is adopted. The method relies on the selection of the most significant coefficients of the PC expansion, allowing a reduction in the number of model evaluations, which are required to build the experimental design for the coefficient estimation.

Following the previously described non-intrusive method, a set of $N^{PCE}<<N$ samples of the input variable θ must be drawn from p(θ), and the corresponding model responses v are numerically computed through the model M(θ,d). Once the metamodel $M^{PCE}$ is built, the *N* samples required for the estimation of $\hat{U}\left(d\right)$ can be computed through the surrogate. The number $N^{PCE}$ of input–output samples needed to build such a surrogate model should be chosen by considering the required accuracy of the metamodel in predicting the response of the original model.

According to the adopted formulation for surrogate modeling, $n_{dof}$ PCE surrogates would be required (see Equation (24)), thus making the computation unbearable. Dimensionality reduction strategies are further required to overcome this computational issue. Principal component analysis (PCA) offers a statistical tool for handling large datasets, first introduced by Pearson [52] and Hotelling [53], and later developed in [54,55] for different fields of application: Karhunen–Loeve decomposition (KLD) [56,57] in signal processing, proper orthogonal decomposition (POD) [58] in mathematics, and singular value decomposition (SVD) in mechanical engineering [59]. Some examples of the application of POD in structural health monitoring can be found in [60,61,62,63], where the method has been employed for the order reduction of dynamical models feeding Bayesian updating schemes.

PCA allows the computational burden to be reduced according to the following procedure. Let the model parameters (i.e., the input variables of the surrogate) be sampled from the prior pdf: $\theta_{i}∼p\left(\theta \right)$, with  $i=1,\ldots ,N^{PCE}$. Compute the response vectors, which are instead the output variable of the surrogate, as $v_{i}=v\left(\theta_{i}\right)$ through the full-order numerical model, building the so-called experimental design of the surrogate model. The model response data are gathered in the matrix $V=\left[\begin{array}{ccc} v_{1} & \cdots & v_{N^{PCE}} \end{array}\right]\in R^{n_{dof}\times N^{PCE}}$. V is projected onto a new space of $n_{dof}$ of uncorrelated variables:

(26) T=WV,

where $W\in R^{n_{dof}\times n_{dof}}$ is a square orthogonal matrix, whose rows are the eigenvectors of the matrix $V^{T}V$ and form an orthogonal basis; $T\in R^{n_{dof}\times N^{PCE}}$ is the matrix of the principal component scores (i.e., the representation of V in the principal component space).

The dimension of the response matrix is reduced through the PCA by retaining only the first $l<<n_{dof}$ components in the solution:

(27) $$T_{l}=W_{l}V,$$

where $T_{l}\in R^{l\times N^{PCE}}$ is the reduced-order response matrix and $W_{l}\in R^{l\times n_{dof}}$ is orthonormal.

The formulation defined in Equation (24) is then modified by setting the output of the PCE surrogate model as the reduced-dimension response vector, thus establishing a relation between the parameters θ and the first principal components of v.

In conclusion, by combining the PCE surrogate and the PCA dimensionality reduction technique, the original model response M(θ,d) can be approximated through:

(28) $$M\left(\theta ,d\right)≅M^{META}\left(\theta ,d\right)=L\left(d\right){W_{l}}^{T}M^{PCE}\left(\theta \right),$$

where $M^{META}\left(\theta ,d\right)$ is the PCA-PCE-based metamodel.

The resulting MC estimator then reads:

(29) $$\begin{array}{c} \hat{U}\left(d\right)=\frac{1}{N}\sum_{i=1}^{N}\left\{\ln\left[p_{\epsilon }\left(\epsilon^{i}\right)\right]\right\}-\\ -\frac{1}{N}\sum_{i=1}^{N}\left\{\ln\left[\frac{1}{N}\sum_{j=1}^{N}p_{\epsilon }\left(L\left(d\right){W_{l}}^{T}M^{PCE}\left(\theta^{i}\right)+\epsilon^{i}-(L\left(d\right){W_{l}}^{T}M^{PCE}\left(\theta^{j}\right)\right)\right]\right\}, \end{array}$$

where, according to the formulation presented, the design variable d that defines the spatial configuration of the sensor network is defined as follows:

(30) $$d=\left\{\begin{array}{c} d_{1}\\ \vdots \\ d_{s}\\ \vdots \\ d_{n_{y}} \end{array}\right\},$$

where:

(31) $$d_{s}={\left\{{x_{1}}^{s}\;\;{x_{2}}^{s}\;\;{x_{3}}^{s}\;\;\delta_{s}\right\}}^{T},$$

and ${x_{1}}^{s},\;{x_{2}}^{s},\;{x_{3}}^{s}$ are the coordinates of the location where the *s*-th measurement is supposed to be taken. $\delta_{s}$ is a scalar integer value which defines the measured DOF—either a displacement or a rotation.

A possible alternative formulation relies on the nodal labeling of the numerical model DOFs. Despite the beneficial dimension reduction that can be reached, the adoption of this formulation would be detrimental in the solution of the optimization problem, as it would lead to consistent discontinuities of the objective function in the associated search space.

## 5. Optimization Procedure

The proposed procedure for optimal sensor placement is based on the estimation of the expected Shannon information gain U(d) through the MC estimator $\hat{U}\left(d\right)$.

According to [7], a sequential strategy can be adopted to solve the problem. At each iteration, only the position of one sensor is optimized, while all other sensors deployed in the previous algorithm steps are held fixed. This strategy was termed forward sequential sensor placement. On the contrary, with the backward sequential sensor placement strategy, the initial configuration is populated with sensors at all of the nodes, and they are later dropped from the optimal configuration one-by-one. In this regard, such sequential strategies are expected to yield sub-optimal solutions, since they cannot guarantee that the optimal solution (i.e., the global maximum of the objective function) is attained. Independently of the method adopted, the iterations are stopped when the desired number $n_{y}$ of sensors are placed over the structure.

Since the estimator $\hat{U}\left(d\right)$ is based on Monte Carlo sampling of measurement error ϵ∼$p_{\epsilon }$ and parameters vector θ∼p(θ|d), the resulting objective function becomes noisy. As discussed in Section 2.1, the prior pdf can be assumed to be independent of the position of the sensors (i.e., θ∼p(θ|d)=p(θ)). The same batch of samples θ can be used for each realization of d, resulting in a less-noisy objective function. It should be underlined that, following this assumption, the objective function will be affected by a constant bias, which therefore will not influence the resulting optimal solutions in terms of sensor configuration. Moreover, since there is no need to re-sample θ and compute the corresponding structural response for each different sample of d at each iteration of the optimization procedure, a consistent reduction in the overall computational cost is achieved.

Due to the noisy objective function, in order to avoid the attainment of a false local optimum, the covariance matrix adaptation evolution strategy (CMA-ES) [64] is adopted here. It is an iterative evolutionary derivative-free algorithm that is suitable for stochastic optimization problems, introduced in [65,66].

The pseudo-code of the CMA-ES is listed in Algorithm 1. The algorithm is based on an evolutionary strategy, where at each iteration *i*, a total number $N^{opt}$ of samples d are drawn from a multivariate normal distribution $d_{j}$∼$m+\sigma_{c}N\left(0,C\right)$, where $C\in R^{n_{d\times d}}$ is the covariance matrix, $m\in R^{n_{d}}$ is the mean of the design points distribution, and $\sigma_{c}$ is the step size. Then, the values of m, C, and $\sigma_{c}$ are updated in order for the population of new points $d_{1:N^{opt}}$ to move towards the maximum of the objective function $\hat{U}\left(d\right)$. The evolution of the design variable (i.e., the sequence of consecutive steps of the mean m) is performed through the so-called cumulation technique, detailed in Algorithm 1, moving from the initial condition $d_{0}$, and μ and $\mu_{w}$ are parameters needed to control the update phase. In Algorithm 1, $c_{c}$, $c_{\sigma_{c}}$, $c_{1}$, $d_{\sigma_{c}}$ are parameters which control the optimization procedure and have to be set empirically for each numerical application. The iterations are stopped whenever at least one of the following criteria are fulfilled:

(32) $$\left\{\begin{array}{c} \;\left|\hat{U}\left(d_{k}\right)-\hat{U}\left(d_{k-1}\right)\right|\leq \rho_{U},\\ \;∥d_{k}-d_{k-1}∥\leq \rho_{d}, \end{array}\right.$$

where the symbol $\left|\cdot \right|$ stands for the absolute value of the argument; $∥\cdot ∥$ represents an appropriately chosen norm of vectors (e.g., the $L^{2}$ norm used here); and $\rho_{U}$ and $\rho_{d}$ are parameters that tolerances the accuracy of the solution in terms of objective function and design variable, respectively. These parameters cannot be chosen a priori, as they are dependent on the specific application, on the model discretization, and on the desired accuracy. For further details on the algorithm, the interested reader may refer to [16,67].

**Algorithm 1** Covariance matrix adaptation evolution strategy

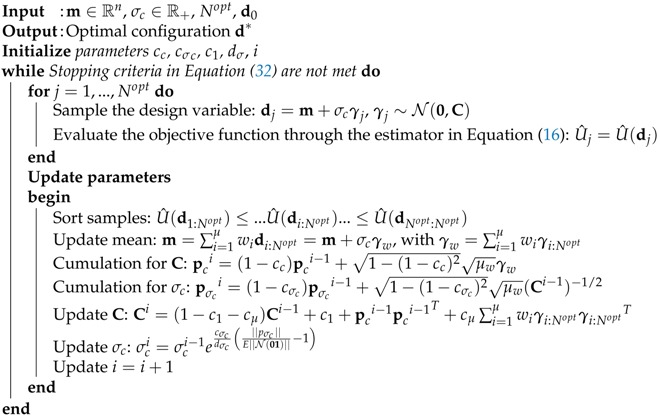



**Figure 1:**
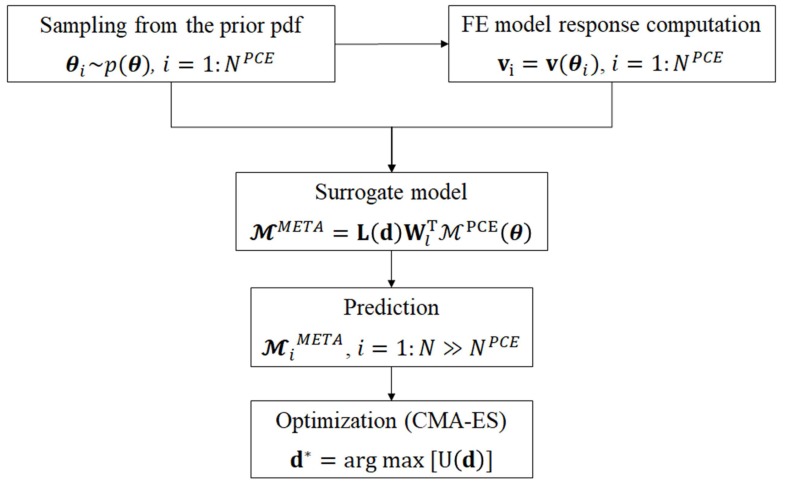
Flowchart of the proposed procedure. CMA-ES: covariance matrix adaptation evolution strategy; FE: finite element.

The overall procedure for computing the optimal sensor configuration is listed in Algorithm 2, and the corresponding flowchart is shown in Figure 1. First, the parameter vector θ is sampled from the prior pdf p(θ), which is chosen a priori. For each sample $\theta_{i}$ (with $i=1,\ldots ,N^{PCE}$), the corresponding response $v_{i}=v\left(\theta_{i}\right)$ is computed through the numerical model. Then, the dimension of the response vector is reduced from $n_{dof}$ to *l* by performing the PCA of $V=[v_{1}\;\cdots \;v_{N^{PCE}}]$. A total number *l* of model surrogates is built by considering θ as the input variable and the components of the reduced-space vector $T_{l}$ as the output variables. A fresh batch of $N>>N^{PCE}$ samples θ is drawn from prior p(θ), and the corresponding system response is computed through the PCE surrogates. In the end, the optimal configuration is obtained through the CMA-ES optimization method listed in Algorithm 1, where the evaluation of the objective function is performed through the MC estimator defined in Equation (16).

**Algorithm 2** Algorithm for the optimization of SHM sensor networks through Bayesian experimental design

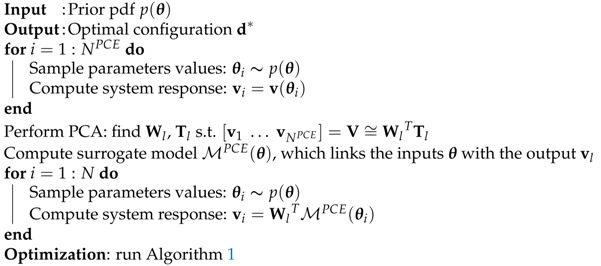



## 6. Results: Application to the Monitoring of a Tall Building

**Figure 2:**
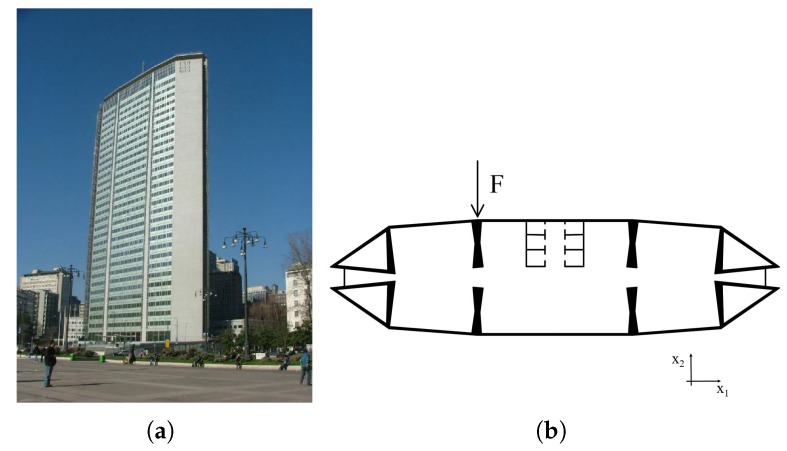
Structural details of the Pirelli Tower: (**a**) 3D view and (**b**) plan representation.

The framework described in the present work was applied to a real large-scale structure: the Pirelli Tower, a 130-m-tall building in Milan (Italy). The building consists of 35 stories out of ground, which are approximately 70 m long and 30 m wide (see Figure 2). The structural system is entirely made of reinforced concrete. Four symmetric triangular cores at the two extremities of each storey are connected by T-shaped beams (see Figure 2b). The structure was modeled using the commercial software SAP2000 v19 (Computer and Structures, Inc., Berkeley, CA, USA) and the associated finite element (FE) model, shown in Figure 2a, consists of 4106 nodes with 6 DOFs each (the 3 displacements $u_{x_{1}}$, $u_{x_{2}}$, $u_{x_{3}}$, and the 3 rotations $\varphi_{x_{1}}$, $\varphi_{x_{2}}$, $\varphi_{x_{3}}$ about the axes of the reported orthonormal reference frame), resulting in a total number of DOFs $n_{dof}$ = 24,500. The structure is supposed to be subjected to a horizontal force acting in the $x_{2}$ direction on the top floor (Figure 2b), see also [14,38,68,69]. The force was assumed to be eccentric, as shown in Figure 2b, in order to induce a complex bending-torsional mechanical response of the tower. For further details on the structural characteristics and on the FE model, the readers may refer to [68,70,71].

The sensor network was assumed to be optimized in terms of the $n_{\theta }=6$ parameters, which are listed in Table 1. The parameters were chosen to render the example as general as possible. Both mechanical and geometrical properties are handled, associated to both vertical and horizontal structural members. The chosen parameters were the Young’s moduli of column groups LC and RC, the Young’s moduli of beam groups LB, CB, and RB, and the beam thickness of group CB. The prior pdfs of each parameter are also shown in Table 1. The prior pdfs of the concrete Young’s modulus were assumed to be uniform, with lower and upper bounds respectively equal to 24GPa and 36GPa. The prior pdf of the beam thickness was considered to be uniform as well, with lower and upper bounds respectively equal to 0.7m and 0.9m.

Since the structural model features both displacement and rotation DOFs at each node, the design variable d must be defined such that both the spatial position of the sensors and the physical quantity to be measured are taken into account, in accordance with Equations (30) and (31).

As discussed in Section 2.2, in the optimization procedure it is assumed that only the standard deviation σ associated with the measurement error can be varied, while the model error is supposed to be a constant term. As σ is assumed to be dependent on the sensor characteristics, we also aim to provide a procedure which allows the optimal type of sensor to be chosen to better estimate the chosen parameters, such as possible variations of the estimated properties from the initial health state of the structure.

**Figure 3:**
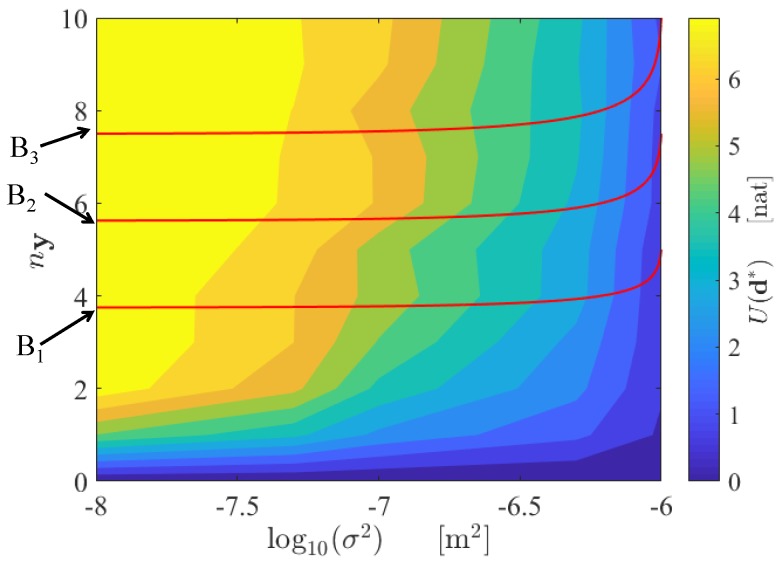
Contour plot of $\bar{U}\left(n_{y},\sigma \right)$, and curves representing the budget constraints $B=C(\sigma ,n_{y})$, with $B_{1}=2000\;€$, $B_{2}=2500\;€$, $B_{3}=3000\;€$ .

**Figure 4:**
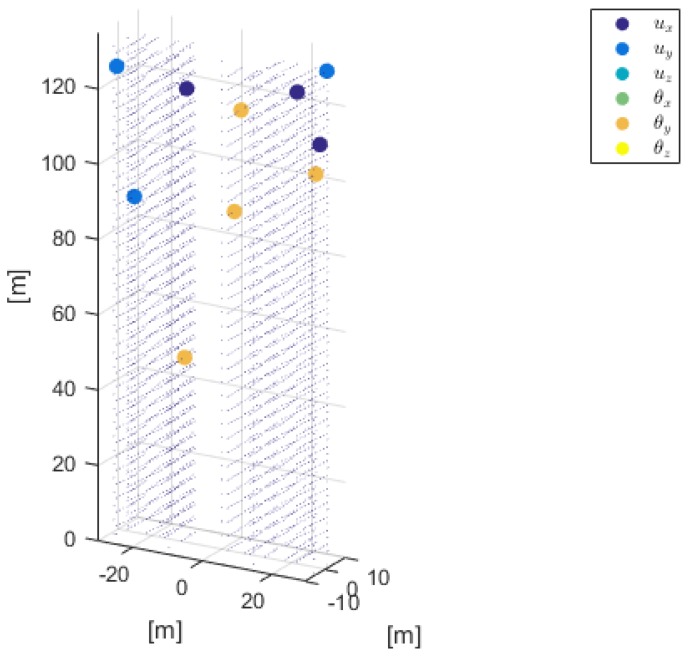
Optimal sensor placement and physical quantity to be measured, with $n_{y}=10$ and $\sigma =10^{-8}$.

The contour plot of the objective function $\bar{U}\left(n_{y},\sigma \right)=U\left(d^{*},n_{y},\sigma \right)$ is shown in Figure 3. Here, the objective function is computed at the following discrete points of the grid $\left(n_{y},\sigma^{2}\right)=\left\{1,2,3,4,5,6,7,8,9,10\right\}\times \left\{10^{-8},10^{-7.5},10^{-7},10^{-6.5},10^{-6}\right\}$. As expected, the maximum value of the expected Shannon information gain increases as the number of sensors increases, as analytically proven in [6] and numerically shown in [72], while the standard deviations decreases, since more information is provided by the SHM system. The associated optimal sensor configuration, which corresponds to the maximum of the objective functions, is shown in Figure 4. A further discussion of the optimal sensor placement with this method can be found in [43].

It can be also observed that the increase in the expected Shannon information gain decreases as more measurements are allowed for. The quantity $\frac{\partial U}{\partial n_{y}}$ is therefore a decreasing function of $n_{y}$. From a decision-making perspective, it is interesting to underline that this behaviour corresponds to the so-called “law of diminishing marginal utility”, also known as Gossen’s First Law [73], which is used in economics for the optimization of resource allocation. The law states that the marginal utility of each unit decreases as the supply of units increases. In the problem of optimal SHM system design, the utility of the sensor network is quantified by the expected Shannon information gain [18], and the unit is represented by each measurement. Applications of this law to sensor network optimization in different engineering fields can also be found in [74,75,76].

As a simple linear cost model is assumed (see Equation (10)), the red lines in Figure 3 represent different budget constraints (i.e., the solutions $\left\{\sigma \;n_{y}\right\}$ of the equation $B=C_{0}+c\left(\sigma \right)\;n_{y}$, where *B* is the total available budget). By using this chart, it is possible to optimally design the SHM network: the optimal point $\left\{\sigma^{*}\;n_{y}^{*}\right\}$, for which $\bar{U}\left(n_{y},\sigma \right)$ is maximum, is ruled by the available budgetary constraint and it is uniquely associated with the corresponding optimal configuration $d^{*}$.

**Figure 5:**
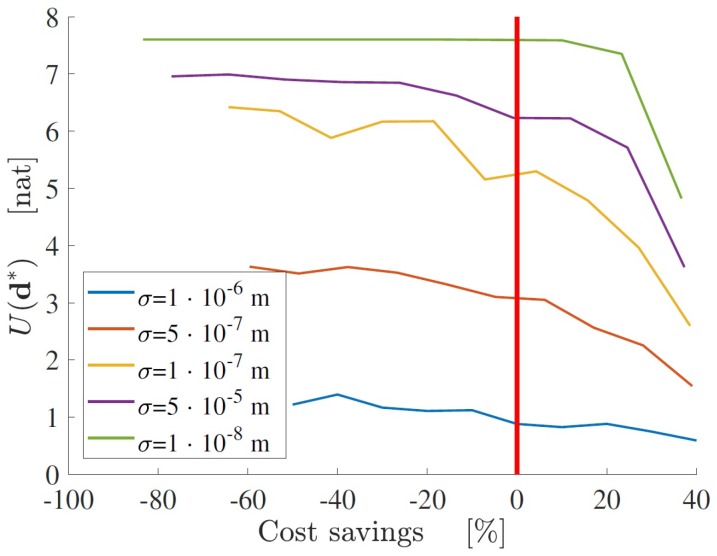
Pareto fronts of the structural health monitoring (SHM) sensor network optimization problem, for different values of standard deviation σ.

A different approach for decision-making is to define a Pareto-like graph, as shown in Figure 5. Each line corresponds to the optimal design for a certain standard deviation (i.e., a certain type of sensors). The cost saving is defined in order to normalize the cost function with respect to the chosen budget. Accordingly, any solution point located at the left of the vertical line represents a non-optimal design solution, since the associated cost does not correspond to the best choice of $\left\{d,n_{y},\sigma \right\}$. The oscillations of the objective functions in Figure 5 are due to the possible presence of local maxima. Although this problem cannot be solved a priori, it can be mitigated by running the optimization algorithm several times with different initial points $d_{0}$, and choosing the optimal configuration which corresponds to the maximum value of the objective function among the correspondent solutions.

This graph can be particularly useful in appropriately allocating economic resources. For a chosen budget, it is possible to select the type of sensors which results in the highest accuracy as indicated in Figure 5, the number of sensors, and their location, associated with the maximum possible expected information gain. The trend of each Pareto front provides an indication about the change of maximum utility due to a variation of budget, and thus it helps to decide if additional spending is justified. Moreover, given the value of *U*, it is possible to compare, from an economic point of view, different solutions in terms of the number and type of sensors.

**Figure 6:**
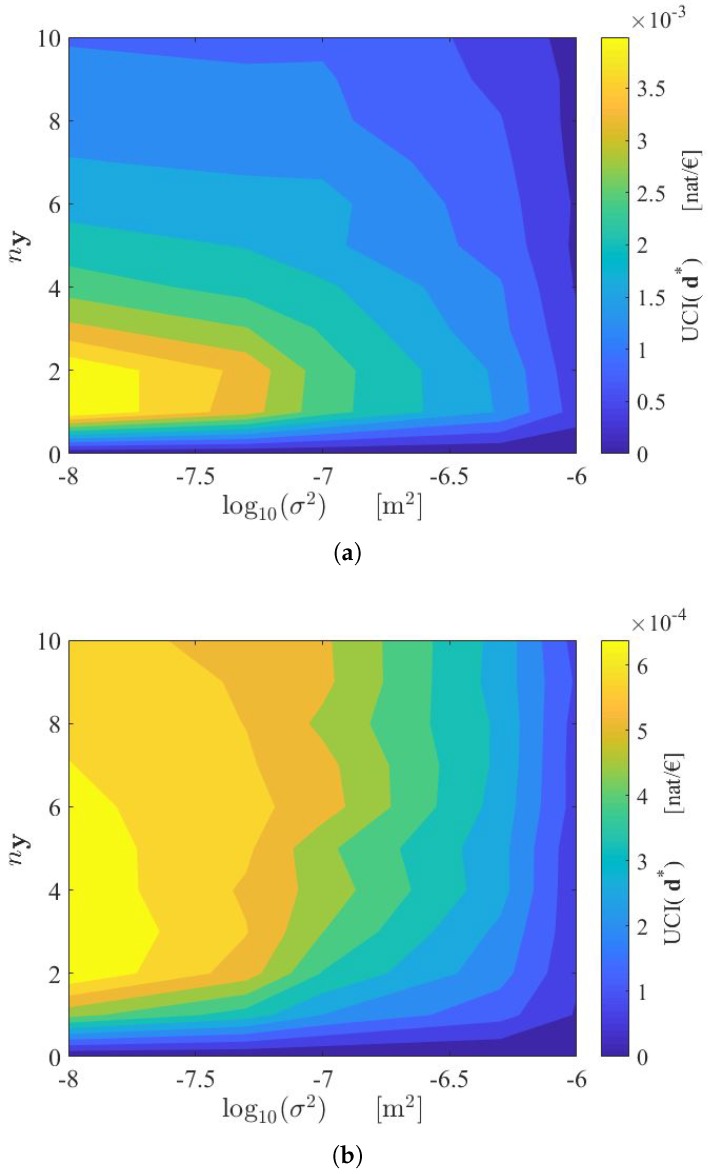
Contour plot of $UCI\left(n_{y},\sigma \right)=\frac{\bar{U}\left(d^{*},n_{y},\sigma \right)}{C(n_{y},\sigma )}$, with (**a**) $C_{0}=500\;€$ and (**b**) $C_{0}=1000\;€$. UCI: utility–cost index.

An alternative design approach discussed in Section 2.2 with Equation (11) is based on the maximization of the ratio $UCI\left(n_{y},\sigma \right)=\frac{\bar{U}\left(d^{*},n_{y},\sigma \right)}{C(n_{y},\sigma )}$. The resulting optimal solution depends on the cost model. In Figure 6a the SHM system is supposed to have a low initial cost (i.e., $C_{0}=500\;€$ ). In Figure 6b the SHM system is supposed to have a high initial cost (i.e., $C_{0}=1000\;€$ ). In both cases, the most efficient employment of resources is reached if the best sensor in terms of measurement noise is chosen, while the optimal number of sensors depends on the cost model.

Note that while the objective function $\bar{U}\left(n_{y},\sigma \right)$ always increases with $n_{y}$ and σ, the function $UCI(n_{y},\sigma )$ presents a maximum for $n_{y}<\infty$. This is because, as previously discussed, the increase in information associated with each additional sensor decreases as more sensors are added to the monitoring system. From a cost–benefit point of view, it is therefore worthless to add sensors (i.e., to increase the SHM cost) if the resulting benefit in terms, for example, of the additional expected Shannon information gain is very low.

## 7. Conclusions

The present paper presents a stochastic cost–benefit methodology to optimally design structural health monitoring systems.

The benefit or usefulness of the SHM system is quantified through the expected Shannon information gain between the prior and the posterior pdfs of the parameters to be estimated. By maximizing this objective function, it is possible to choose the best position, type, and number of sensors, which guarantees the minimization of the uncertainties associated with the quantities to be estimated, or in other words, the maximization of the information obtained through the measurements.

The objective function can be numerically approximated through a Monte Carlo sampling approach. The resulting estimator is expressed as a double sum of terms, which depend on the likelihood function. Since a high number of model response evaluations is required, a procedure based on surrogate models and model order reduction strategies is proposed. The combination of the PCE surrogate model and a model order reduction technique (PCA) allows a computationally efficient meta-model to be built in order to mimic the relation between input and output variables. Since the resulting objective function is affected by noise, leading to possible undesired local maxima, an evolutionary strategy (CMA-ES) suitable for stochastic problems is used.

In order to find the optimal solution, the cost, identifiability of the model parameters, and technological constraints have to be taken into account. A further optimization problem is considered, established by maximizing the information gain per unitary cost by means of a cost–benefit analysis.

Application of the framework to a large-scale numerical model demonstrates that the maximum expected Shannon information gain of the SHM system increases as more sensors are added to the system and lower standard deviations of the prediction error are considered. The optimal solution, in terms of maximum information gain, does not necessarily correspond to the most efficient one (see Figure 3), in terms of the ratio between information and cost. This is because the increase in information gain due to additional sensors is reduced as more measurements are considered. A Pareto-front approach can also be followed in order to choose the best solution, both in terms of maximum information and minimum cost (Figure 5).

An alternative procedure based on the maximization of the utility–cost ratio can be implemented to optimally allocate the available resources. In this case, the optimal solution depends on the variation of the sensor network cost with respect to the number of measurements and the sensor type (see Figure 6a,b). It is worth noting that the same consideration also holds for the case where only a few types of sensors are available, and therefore if it is not possible to establish a cost model. The optimization can be performed in the same way. That is, by computing the maximum values of the objective function (which correspond to the optimal spatial configurations) over the discrete search grid.

The proposed strategy is completely non-intrusive, in that it does not require computation of the gradient of the objective function, but instead exclusively relies on evaluations of model response. Moreover, the method is general and no restrictive assumptions, such as linearity or Gaussianity, are placed.

Further work will be dedicated to the application of this framework to dynamic testing and to more complex structural models.

## Figures and Tables

**Table 1:** Definition of parameters θ (see Figure 2) and related prior probability density function (pdf) p(θ).

Position	Physical Quantity	Prior pdf
20th floor left columns (LC)	Young’s modulus *E* (GPa)	U(24,36)
20th floor right columns (RC)	Young’s modulus *E* (GPa)	U(24,36)
20th floor left beams (LB)	Young’s modulus *E* (GPa)	U(24,36)
20th floor right beams (RB)	Young’s modulus *E* (GPa)	U(24,36)
20th floor central beams (CB)	Young’s modulus *E* (GPa)	U(24,36)
20th floor central beams (CB)	Beam thickness *t* (m)	U(0.7,0.9)

## Abbreviations

The following abbreviations are used in this manuscript:

SHM	Structural Health Monitoring
PCE	Polynomial Chaos Expansion
PCA	Principal Component Analysis
CMA-ES	Covariance Matrix Adaptation Evolutionary Strategy
KLD	Kullback–Leibler Divergence
UCI	Utility-Cost Index
MC	Monte Carlo
MI	Mutual Information
DOF	Degree of Freedom
FE	Finite Element
